# Accurate Calculation of FFR Based on a Physics-Driven Fluid‐Structure Interaction Model

**DOI:** 10.3389/fphys.2022.861446

**Published:** 2022-04-12

**Authors:** Xiaolu Xi, Jincheng Liu, Hao Sun, Ke Xu, Xue Wang, Liyuan Zhang, Tianming Du, Jian Liu, Bao Li

**Affiliations:** ^1^ Department of Biomedical Engineering, Faculty of Environment and Life, Beijing University of Technology, Beijing, China; ^2^ Cardiovascular Department, Peking University People’s Hospital, Beijing, China

**Keywords:** coronary artery, fluid-structure interaction, 0D/3D geometric multi-scale model, fractional flow reserve, hemodynamic effects

## Abstract

**Background**: The conventional FFRct numerical calculation method uses a model with a multi-scale geometry based upon CFD, and rigid walls. Therefore, important interactions between the elastic vessel wall and blood flow are not routinely considered. Changes in the resistance of coronary microcirculation during hyperaemia are likewise not typically incorporated using a fluid–structure interaction (FSI) algorithm. It is likely that both have resulted in FFRct calculation errors.

**Objective**: In this study we incorporated both the influence of vascular elasticity and coronary microcirculatory structure on FFR, to improve the accuracy of FFRct calculation. Thus, in this study, a physics-driven 3D–0D coupled model including fluid–structure interaction was established to calculate accurate FFRct values.

**Methods**: Based upon a novel geometric multi-scale modeling technology, a FSI simulation approach was used. A lumped parameter model (0D) was used as the outlet boundary condition for the 3D FSI coronary artery model to incorporate physiological microcirculation, with bidirectional coupling between the two models.

**Results**: The accuracy, sensitivity, specificity, and both positive and negative predictive values of FFR_DC_ calculated based upon the coupled 3D–0D model were 86.7, 66.7, 84.6, 66.7, and 91.7%, respectively. Compared to the calculated value using the basic CFD model (MSE = 5.9%, accuracy rate = 80%), the FFR_CFD_ calculated based on the coupled 3D–0D model has a smaller MSE of 1.9%.

**Conclusion**: The physics-driven coupled 3D–0D model that incorporates fluid–structure interactions not only consider the influence of the elastic vessel wall on blood flow, but also provides reliable microvascular resistance boundary conditions for the 3D FSI model. This allows for a calculation that is based upon conditions that are closer to the physiological environment, and thus improves the accuracy of FFRct calculation. It is likely that more accurate information will provide an enhanced recommendation regarding percutaneous coronary intervention (PCI) in the clinic.

## Introduction

The hemodynamic environment inside coronary arteries significantly affects the abnormal growth of vascular endothelial cells and the deposition of cellular lipids, leading to the formation of vascular stenosis, which plays a key role in a heart attack (Bentzon et al., 2014). In the past few decades, hemodynamic studies on coronary artery stenosis have shown that arterial stenosis will severely disrupt normal blood flow, and that blood flow disorder can accelerate the growth of plaque to form a more stable stenosis. Fractional flow reserve (FFR), defined as the maximum myocardial ratio, i.e., the ratio of the blood flow of the stenotic branch of the coronary artery to the blood flow of the same coronary artery, is the current ‘gold standard’ for diagnosing functional myocardial ischemia (Pijls et al., 1996; Kakouros et al., 2013; Pijls et al., 2013; Van De Hoef et al., 2013). When small blood vessels in the coronary blood supply have a maximal dilation and the central venous pressure is assumed to be negligible, FFR can be approximated as:

$$\text{FFR}=\frac{p_{\text{d}}}{p_{\text{a}}}$$ (1)

where *p*_a_ and *p*_d_ are respectively the average pressure of the aortic root and the distal portion of the stenotic coronary artery in the maximum hyperemia state.

Based upon computational fluid dynamics (CFD), FFRct (Fractional Flow Reserve derived from non-invasive coronary CT angiography) was first proposed by Taylor and co-workers (Taylor et al., 2013), who used a computerized numerical simulation to non-invasively calculate FFR. The study coupled lumped parameter models of the heart, systemic circulation, and coronary microcirculation to patient-specific models of the aortic root and epicardial coronary arteries reconstructed from data acquired from computed tomography angiography (CTA). Therefore, a geometric multi-scale model of the coronary artery was established to realize the non-invasive FFR calculation. The research team led by C. A. Taylor conducted the largest FFRct study in the world, representing the highest level of research on FFRct (Taylor et al., 2013; Zarins et al., 2013). After proposing the above-mentioned FFRct calculation method, they successively carried out three large-scale research projects: DISCOVER-FLOW, DeFACTO, and HeartFlowNXT. Clinical experiments proved that FFRct can accurately diagnose and rule out coronary stenosis causing myocardial functional ischemia. Recently the Heart Flow-funded PLATFORM study (Prospective Longitudinal Trial of FFRct: outcome and resource impacts) further demonstrated the effectiveness of FFRct in the clinical diagnosis of myocardial ischemia: compared with CTA, FFRct can significantly reduce the false positive rate in patients with coronary heart disease. (Grunau et al., 2013; Douglas et al., 2015). There are now studies pursuing fast numerical calculation of FFR. For instance, Zhang et al. (2016) used a simplified steady-state coronary flow model for non-invasive calculation of FFR. However, the studies ignored the elasticity of blood vessels based upon a single-coupled numerical calculation model. The model assumed that the vessel wall is rigid, resulting in inaccurate numerical simulation.

Human blood vessels are elastic, and pulsating blood flow presses upon the blood vessel wall in real time, causing the deformation of the blood vessel wall and a change in the flow field. Therefore, numerical simulation using a multi-scale CFD model with a fixed geometry will ignore the real-time influence of the blood vessel wall on blood flow. The hypothesis of a rigid wall cannot be used to reflect real hemodynamics within blood vessels, and this may bias FFRct calculation. The dilation of blood vessels and plaques in an elastic wall will cause a gap between true vascular resistance and that of the rigid wall, which leads to significant differences in calculated FFRct, and potentially a false-negative diagnosis (Kock et al., 2008; Tang et al., 2009; Teng et al., 2010).

In addition to the presence of vascular elasticity, the influence of microcirculation resistance on calculated FFRct should not be ignored. The fluid‐structure interaction (FSI) analysis of blood flow and the blood vessel wall can consider the mechanical interaction between blood flow and the blood vessel wall (Perktold and Rappitsch, 1995; Tang et al., 2003), thereby reducing calculation errors. However, the traditional FSI model cannot fully consider the changes in microcirculation resistance of coronary arteries in a state of hyperemia. The total resistance of coronary arteries is related to coronary microcirculation, and myocardial blood flow is regulated by coronary microcirculation (Leung and Leung., 2011). Microcirculation is an important part of the circulatory system, which plays an irreplaceable role in promoting cardiovascular health. For a more accurate model incorporating microcirculation, a flow analysis of the circulatory system should be performed (He et al., 2021). To fully consider patient-specific microcirculation resistance, a geometric multi-scale model should be established to non-invasively calculate FFR (Lagana et al., 2005; Kim et al., 2010; Moghadam et al., 2013; Zhao et al., 2015).

Based on the rule of energy conservation, stenosis and microcirculation resistance should fit the following formula:

$$p_{\text{a}}=\text{Δ}p_{\text{stenosis}}+\Delta p_{\text{micro}-\text{circulation}}+p_{\text{v}}$$ (2)

where *p*_a_ is the aortic pressure, $\Delta p_{stenosis}$ is the pressure drop of stenotic vessels, $\Delta p_{micro-circulation}$ is the microcirculation pressure drop, and $p_{v}$ is the right atrial pressure, which is generally small and can be ignored.

In order to consider the influence of both FSI and coronary microcirculation structure on FFR, this study proposes a dual-coupled 3D FSI–0D numerical model (FFR_DC_) for non-invasive calculation of FFR to improve the accuracy of FFRct calculation. In this study, 15 patient-specific CTA images were collected, as well as clinically measured FFR for comparison. FFR_DC_ was non-invasively calculated and compared with the clinically measured FFR to determine the reliability and accuracy of the method for diagnosing myocardial ischemia.

## Methods

### Establishment of Dual-Coupled Three-Dimensional Fluid–Structure Interaction–Zero-Dimensional Model

**FIGURE 1:**
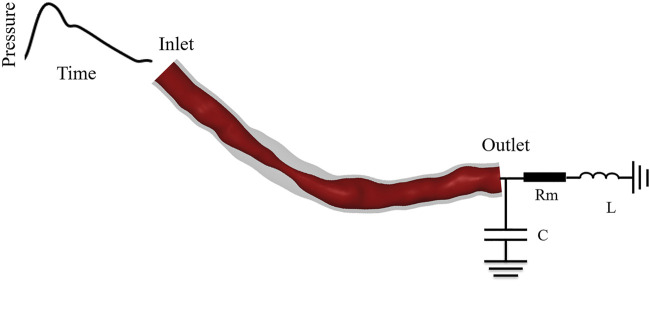
Dual-coupled coronary model with 70% stenosis and 10 mm stenosis length.

In this study, a coupled multi-scale 3D–0D model of the coronary artery incorporating FSI was established by coupling a zero-dimensional (0D) model with a three-dimensional (3D) model coronary using a blood flow domain and vascular structure domain, as shown in Figure 1. The complete model consisted of the coronary artery structure and microcirculation structure. The 3D FSI model described the coronary artery structure, and the 0D model was used to provide the boundary conditions for the coronary artery outlet.

The coronary artery structure described by the 3D model included structural domains and fluid domains. According to the patient’s CTA image, the 3D reconstruction software Mimics was used to reconstruct the fluid domain of the patient-specific epicardial coronary artery, and then the fluid part was shelled to obtain coronary artery structural domains. The calculation of the geometric multi-scale model requires multiple iterative calculations in each time step. To simplify the calculation, the 3D model only included the stenotic segment for calculation, the specific length included followed the formula of Sankaran et al. (2016):

$$\text{d}\left(\text{z}\right)=d_{h}\left(z\right)\left[1-\frac{1}{2}\left[1-\cos\left(\frac{z-z_{\text{c}}}{\Delta }\pi +\pi \right)\right]\alpha \right]z_{\text{t}}<z<z_{\text{u}}$$ (3)

where $d_{h}$ is the normal blood vessel diameter, $z_{c}$ is the position of the smallest diameter, $z_{t}$ and $z_{u}$ are respectively the start and end points of the stenosis, *∆* is half of the length of the stenosis, and *α* is the modeled percentage stenosis.

The 0D model was established based upon similarity between the regulation of electronic circuits and blood flow. A 0D model containing electrical components was used to simulate the cardiovascular system, turning the complex 3D blood flow simulation into a simple circuit simulation (Wischgoll et al., 2008; Huo et al., 2012). Blood flow resistance was simulated by electrical resistance, while blood pressure and blood flow were equivalent to voltage and current. The equivalent relationship between hemodynamic parameters and electrical parameters is described in Table 1. The inductance parameter was set to the empirical value of 0.5, which has been described in detail in previous laboratory studies (Wang et al., 2018), so that the calculation results converged. Resistance values are determined based on physiological parameters such as blood pressure, cardiac output, and coronary branch flow.

**TABLE 1:** Hemodynamic parameters and equivalent electrical parameters.

Hemodynamic parameter	Blood flow	Blood pressure	Microcirculation resistance	Vascular elasticity	Blood flow inertia
Equivalent electrical parameter	Current	Voltage	Electrical resistance	Capacitance	Inductance

### Dual Coupling Algorithm

Data transmission during 3D–0D coupling was performed by user-defined functions (UDF) in ANSYS-Fluent. The inlet boundary condition of the 3D model, $p_{3D,in}$ , is the clinically measured aortic pressure waveform of the patient. The volumetric flow rate, $Q_{3D}$ , is calculated according to the 0D model of microcirculation resistance, *R*_m_, and the 3D FSI model in the hyperemic state determines the outlet pressure

$$p_{3\text{D},\text{out}}=Q_{3\text{D}}\times R_{\text{m}}\times 0.24\;\;$$ (4)

where $p_{3D,out}$ is the outlet pressure of the 3D model, $Q_{3D}$ is volumetric blood flow rate, and $\;R_{m}$ is the microcirculation resistance in the resting state.

The geometric multi-scale model is set up as a transient calculation driven by physics. We calculated FFRct by determining both stenosis resistance and microcirculation resistance. Based upon energy conservation, when Eq 5 is satisfied in a time-step calculation, the 0D model and the 3D model have reached a pressure balance, and the calculation has reached convergence.

$$p_{3\text{D},\text{in}}=\text{Δ}p_{3\text{D}}+p_{3\text{D},\text{out}}$$ (5)

where $\Delta p_{3D}$ is the pressure drop produced in this section of the blood vessel, and $p_{3D,in}$ is the inlet pressure of the 3D model.

FSI provides the calculation of the 3D model, including fluid domain and structure domain, using a two-way fluid–structure interaction method. Blood was set as an incompressible fluid in a 3D, transient simulation. The fluid domain Navier–Stokes equation and momentum equation considering the dynamic mesh algorithm for the numerical calculation of FSI are:

$$\rho_{\text{f}}\left[\frac{\partial v}{\partial t}+\left(v-v_{\text{g}}\right)\cdot \nabla v\right]=-\nabla p+\nabla \cdot T$$ (6)

∇⋅v=0 (7)

where ∇ is the Hamiltonian, ***v*** is the fluid velocity vector, $v_{g}$ is the mesh shifting velocity, ***T*** is the stress tensor, *p* is fluid pressure, and $\rho_{f}$ is fluid density (Bird et al., 1987; Fefferman, 2006; Alireza et al., 2014).

Without considering stress in the calculation, the governing equation of the movement of the vascular wall, namely the solid domain, is:

$$\nabla \cdot \sigma_{\text{s}}=\rho_{\text{s}}\cdot a_{\text{s}}$$ (8)

In the formula, $\sigma_{s}$ is the stress tensor of the vessel wall, $\rho_{s}$ is the density of the vessel wall, and $a_{s}$ is the acceleration of the vessel wall (Fung and Cowin., 1993).

To follow the most basic principles of conservation, the surface that is subject to fluid–solid interaction should also satisfy conservation of stress, displacement, and flow rate of fluid and solid.

$$\sigma_{\text{s}}\cdot {\hat{n}}_{s}=\sigma_{\text{f}}\cdot {\hat{n}}_{f}$$ (9)

$$d_{s}=d_{f}$$ (10)

$$q_{s}=q_{f}$$ (11)

**FIGURE 2:**
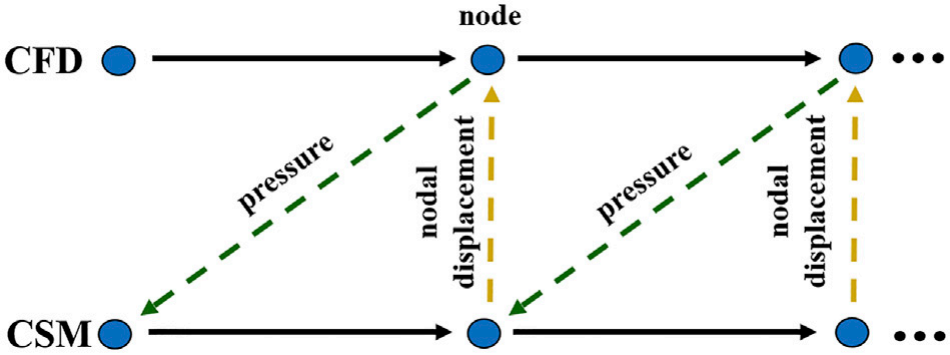
Data transfer between fluid and solid domains. (CFD: computational fluid dynamics; CSM: computational structural mechanics).

In the above formulæ ***d*** is the displacement vector, ***q*** is the flow rate, σ is the stress tensor, $\hat{n}$ is the boundary normal, and the subscripts f and s represent the fluid and solid domains. The data transmission during the bidirectional fluid–solid coupling calculation is shown in Figure 2.

**FIGURE 3:**
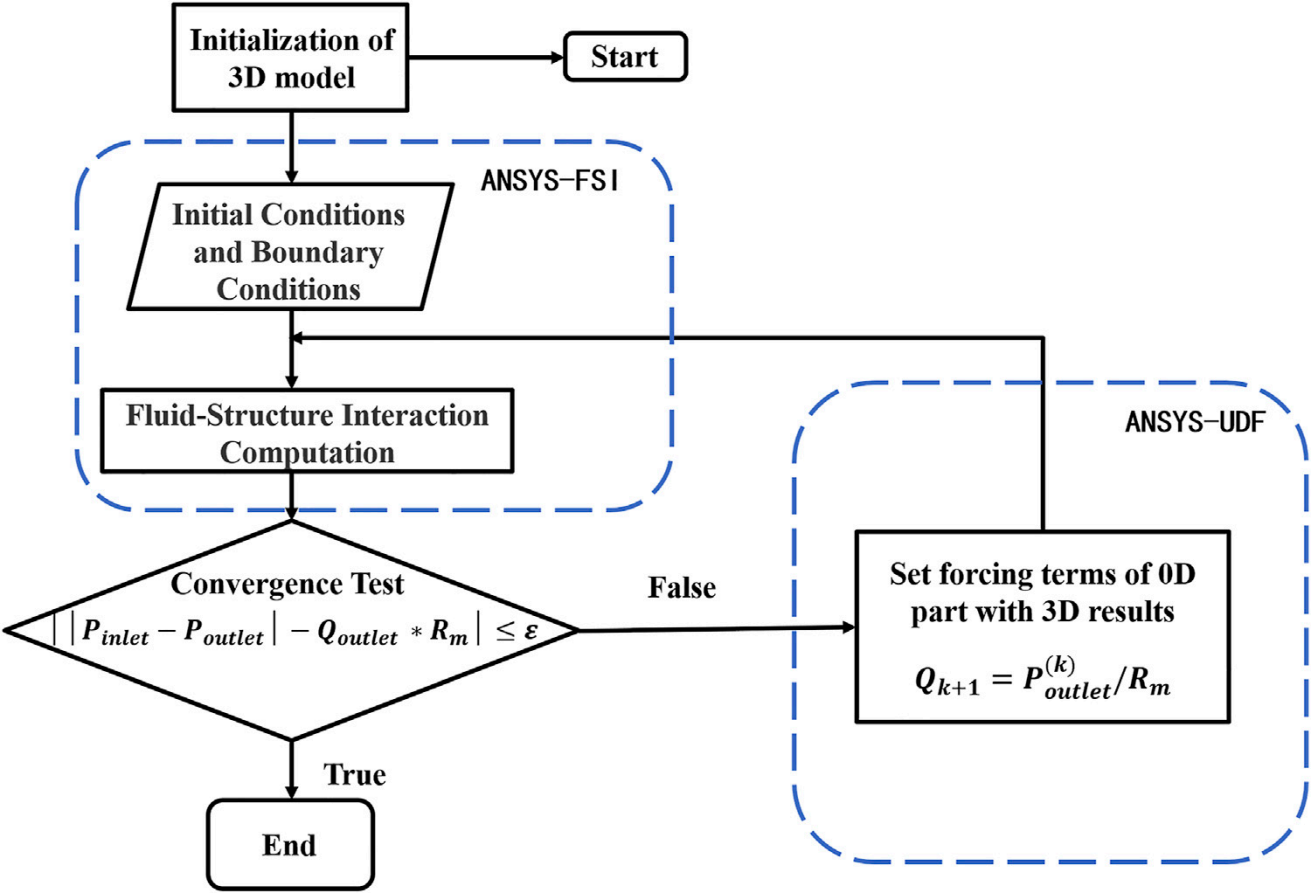
Flow chart of dual-coupled model algorithm.

Based upon the physics-driven method, we implemented two-way coupling between the solid and fluid of the model while also realizing bidirectional coupling between the 0D and 3D models, thus completing the dual coupling model. At each time step of the algorithm, the calculation for the 0-dimensional model was used as the outlet boundary of the 3D model. The inlet pressure of the fluid domain of the 3D model, the displacement of the solid domain, the pressure of the fluid‐solid interface, and the error in displacement data between the fluid and solid domains between different cardiac cycles were defined as the model residuals. When the model residuals were less than the pre-set value, the calculation result was deemed convergent, and then the next calculation proceeded until the end of the simulation. The specific calculation process is shown in Figure 3.

### Simulation of Hyperemia

**FIGURE 4:**
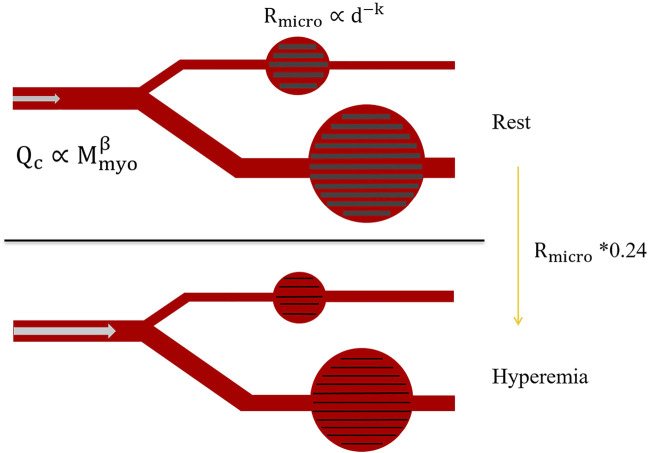
Changes in microcirculation resistance due to hyperemia.

The hyperemia model was obtained by reducing the coronary microcirculation resistance, and it was assumed that all of the patient’s coronary vessels are without stenosis in the rest state. The myocardial mass was calculated by multiplying the reconstructed myocardial volume by the average myocardial density, and the total coronary artery flow was determined by the myocardial mass. According to an allometric scaling law, blood flow is proportional to vessel diameter raised to some power; that is, $Q\propto d^{k}$ (Murray, 1926). The total flow of the coronary vessels was used to calculate the flow of each coronary artery. The microcirculation resistance at the outlet of each branch was calculated according to the formula:

$$R_{\text{m}}=\frac{p}{Q}$$ (12)

where *p* is the coronary branch outlet pressure, and *Q* is the allocated resting flow. Since the FFR was calculated in the hyperemic state, microcirculation resistance was 0.24 times that in the resting state (Wilson et al., 1990; Taylor et al., 2013), as shown in Figure 4.

### Calculation Settings

In this study, uniform material properties were used in the calculation of the vessel wall. It was assumed that the thickness of the blood vessel wall was 0.5 mm (Leach et al., 2010), the arterial wall was non-slip, linear elastic, isotropic and incompressible, the Young’s modulus was 0.6 MPa, and the Poisson’s ratio was 0.48 (Wang et al., 2020). Blood flow was assumed to correspond to incompressible laminar flow of a Newtonian fluid, and the viscosity and density of blood were 0.0035 Pa∙s and 1050 kg/m^3^, respectively (Ofili et al., 1995; Sun et al., 2010). The computation was performed on the ANSYS Workbench platform, solved using a workstation equipped with a 2.3 GHz Intel Xeon CPU and 64 GB of RAM. The systems coupling framework effected the complete coupling of transient fluid analysis (in Fluent CFD) and transient structure analysis. The side of the lumen was set as the interface between fluid and structure. The time steps of the fluid domain and structural domain models were both set to 0.01 s, and the simulation was run for three cardiac cycles. To improve the stability of the coupled simulation, the transmission data value linearly increased in the first five coupling iterations (minimum number of iterations) at each time step. To avoid unstable results of the initial time step, only the data from the second cardiac cycle was used for post-processing. In domains modelled by CFD, the fluid area was divided into tetrahedral elements with inflation layers, and the solid region was divided into a hexahedral grid (Wang et al., 2020).

### Clinical Data Collection and Processing

The study was a prospective investigation. The coronary CTA images were obtained using a dual-layer detector CT system (IQon, Philips Healthcare) comprising 256-row CTA tomography, with a matrix size of 512 × 512 a slice thickness of 0.625 mm, and a pixel size within each slice was 0.5 mm × 0.5 mm. The coronary CTA used standard acquisition protocols in accordance with recommendations from professional societies. All data were collected at the Peking University People’s Hospital, and FFR was measured clinically. The study was approved by the Internal Review Board and informed written consent was obtained from enrolled patients. The anonymous clinical data were independently reviewed and analysed by the Biomechanics Laboratory of Beijing University of Technology.

We set the specific criteria for enrollment, coronary angiography, and FFR catheter surgery. Exclusion criteria for this clinical trial included poor CTA image quality and coronary microcirculation disorders. Acute myocardial infarction (MI), small vascular lesions (defined as reference diameter < 2.5 mm), or N1 vascular lesions were excluded. Clinical data from 15 patients were collected in this study to confirm the accuracy of the dual-coupled model for FFRct calculation.

The dual-coupled calculation was performed for all 15 patients and results were analyzed for statistical significance. Sensitivity, specificity, positive predictive value (PPV), negative predictive value (NPV), and accuracy were expressed as percentages with 95% confidence intervals.

## Results

### Patient Information

Basic information describing the 15 enrolled patients is presented in Table 2. Stenosis was moderate (40–80%), the mean age of patients was 64 years old, and most patients had stable angina and hypertension.

**TABLE 2:** Basic information describing the 15 enrolled patients. Values shown are either counts or mean values (with standard deviations in parentheses, where available).

*Characteristic*	*Value*
Number of patients	15
Number of vessels	15
Ages	64( ± 8.95)
Number of males	10
Number of females	5
Number of left artery descending (LAD)	10
Number of right coronary artery (RCA)	5
Systolic blood pressure/mmHg	140( ± 17.99)
Diastolic blood pressure/mmHg	79( ± 9.01)
Left ventricular systolic volume/mm^3^	31.3( ± 4.3)
Left ventricular diastolic volume/mm^3^	101.84( ± 7.2)
Heart rate/bpm	60.42( ± 8.38)
Cardiac output/(L·min^-1^)	4.251( ± 2.18)
Myocardial mass/g	141.7( ± 23.72)

### FFRct Calculation Results

**FIGURE 5:**
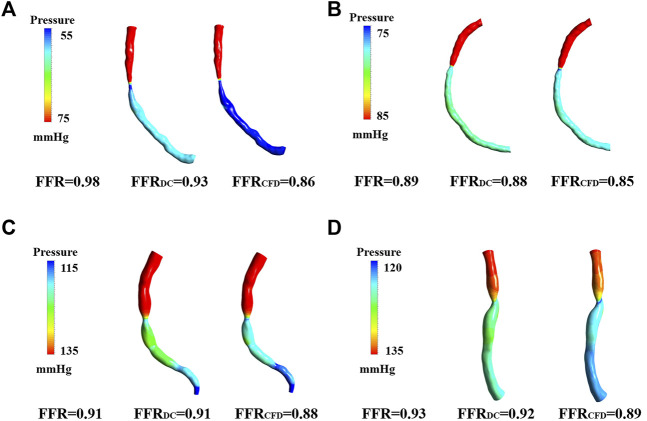
FFRct Pressure cloud image calculated based on dual models. **(A)** Pressure cloud of patient 2 for both computational models. **(B)** Pressure cloud of patient 4 for both computational models. **(C)** Pressure cloud of patient 6 for both computational models. **(D)** Pressure cloud of patient 5 for both computational models.

**FIGURE 6:**
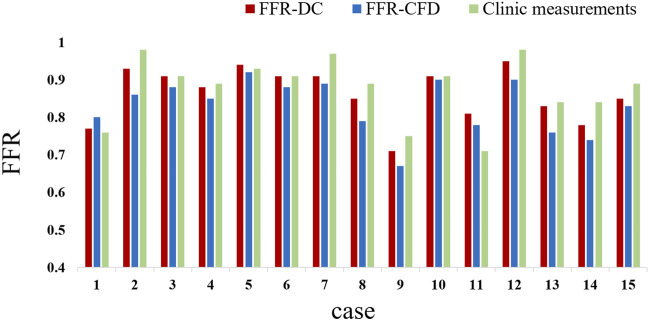
Comparison between clinically measured FFR and FFRct calculated with different models.

Figure 5 shows the FFRct contours calculated using the dual-coupling model and the conventional geometric multi-scale model based upon CFD. Table 3 shows the FFR calculation results for all of the 15 patients. Figure 6 shows the comparison of FFRct calculation results for the 15 patients using the dual-coupling model and the geometric multi-scale model based on CFD: there is a gap between the respective mean FFRct values, demonstrating that the mean value based upon FSI is larger than that based upon CFD alone.

**TABLE 3:** The calculated and measured FFR results for all of the 15 patients.

Patient	Clinically	FFR_DC_	FFR_CFD_	R_m_	Computation time (FFRDC/FFRCFD) [h]
				[mmHg s/ml]	
1	0.76	0.77	0.8	132.99	18/3
2	0.98	0.93	0.86	81.72	9/4.5
3	0.91	0.91	0.88	142.26	10.1/4
4	0.89	0.88	0.85	133.42	12.5/3
5	0.93	0.92	0.89	146.62	10.7/5
6	0.91	0.91	0.88	83.49	11.2/2
7	0.97	0.91	0.89	82.05	8.6/3.3
8	0.89	0.85	0.79	109.27	11.7/4
9	0.75	0.71	0.67	124.17	18.2/3.3
10	0.91	0.91	0.9	427.34	9.5/3
11	0.71	0.81	0.78	143.22	20.4/8
12	0.98	0.95	0.9	95.11	9.1/4
13	0.84	0.83	0.76	101.35	13.7/4
14	0.84	0.78	0.74	105.87	14.6/3.5
15	0.89	0.85	0.83	129.76	12.2/4.3

### Correlation Analysis for Fractional Flow Reserve

When the sample size is constant, the mean square error (MSE) can be used to evaluate the quality of a set of point estimates:

$$\text{MSE}\left(\hat{\theta }\right)=\text{E}{\left(\hat{\theta }-\theta \right)}^{2}$$ (13)

The MSE of FFR calculated based on the dual-coupling model is 1.9%, whilst the MSE of FFR calculated based on the geometric multi-scale CFD model is 5.9%; in each case the reference data were the clinically measured FFR values.

**FIGURE 7:**
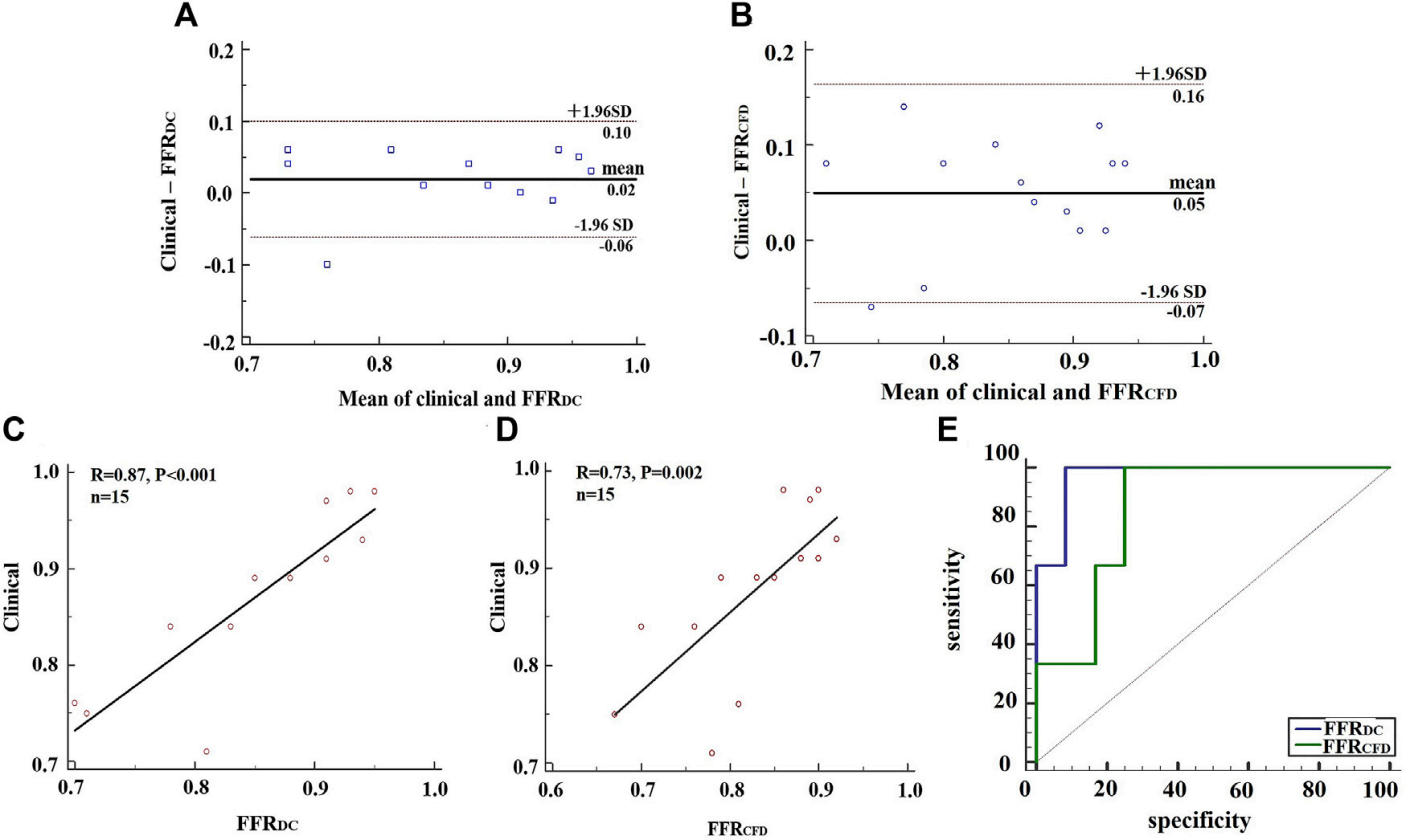
Analysis of clinical FFR data, FFRDC and FFRCFD. **(A)** BlandAltman plots for the pairwise comparisons of clinical FFR data and FFRDC. **(B)** BlandAltman plots for the pairwise comparisons of clinical FFR data and FFRCFD. **(C)** A comparison of clinical FFR data and FFRDC. **(D)** A comparison of clinical FFR data and FFRCFD. **(E)** ROC analysis of FFRDC and FFRCFD, using clinical FFR data as a reference.

The linear relationship between clinically measured FFR and calculated FFRct values is shown in Figure 7 (*p* < 0.01). The Bland–Altman graph and ROC curve between FFR and FFRct are also provided. As shown in the figure, the calculated FFR_DC_ values have a better correlation with the clinically measured values (*R* = 0.87), compared with the values calculated using the conventional CFD model (*R* = 0.73), indicating higher accuracy and diagnostic performance. The ROC curve is a comprehensive indicator reflecting the continuous variables of sensitivity and specificity. AUC (area under the ROC curve) refers to the area under the ROC curve. The closer the AUC is to 1, the higher the diagnostic value of the test. MedCalc 19.20 statistical software was used for ROC analysis. The ROC curves shown in Figure 7E indicate that the AUCs of FFR_DC,_ and FFR_CFD_ are 0.972 (95%CI 0.737–1), *p* < 0.0001 and 0.861 (95%CI 0.589–0.982), *p* = 0.0007. The *p* value for the compare of ROC of FFR_DC,_ and FFR_CFD_ is 0.25. Using FFR ≤ 0.8 as the reference standard, the specificity of FFR_DC_ and FFR_CFD_ is 0.91 (95%CI 0.615–0.998) and 0.75 (95%CI 0.428–0.945) respectively and the sensitivity both are 1 (95%CI 0.292–1). Due to the limitation of quantity, the *p* value of the comparison between the two ROC values indicates that the difference is not significant, but the comparison from AUC reflects the higher accuracy of FFR_DC_. The diagnostic value of FFR_DC_ is higher. The comprehensive results reflect the accuracy of the method for calculating FFRct using the double-coupling model introduced in the present study.

## Discussion

### Influence of the Elastic Wall on FFRct

The FFR value calculated based on the dual-coupling model is 0.03 larger on average than the FFR value calculated by CFD alone. We hypothesize that the reason for that result is the dilation of blood vessel walls, which reduced the resistance to blood flow (Wu et al., 2019). The conventional geometric multi-scale model based on CFD alone assumes that the vessel wall is rigid by default, and there is no change in displacement. Under the same pressure, the stenotic vessel was not deformed in the rigid-walled model, which tended to increase the flow resistance due to stenosis in the vessel. However, blood vessels are elastic during clinical FFR detection, and consequently the FFRct calculation using a rigid-wall model may lead to reduced estimates compared to clinical measurements.

### The Authenticity Based on Use of the Dual-Coupling Model

The FSI model can fully consider the elastic wall, and more closely parallel the physiological state of the human blood vessel. However, the FFRct calculation using conventional FSI cannot fully incorporate the changes in microcirculation resistance after hyperemia. Blood flow within the coronary microcirculation of patients cannot be continuously and non-invasively measured in real time. Therefore we developed and described the physics-driven 3D–0D coupling method. We described boundary conditions and loops at the interface of the modeling domain and specified the 0D model to simulate the resistance of microvessels on the exit boundary conditions of the 3D model to calculate FFRct.

### The Accuracy of the Dual Coupling Model to Calculate FFR_DC_

The Discovery-flow study (Diagnosis of Smaller-Causing Stenoses *via* Noninvasive Fractional Flow Reserve) was an early multi-center, prospective evaluation of FFRct accuracy, which compared FFRct with invasive FFR, showing that FFRct had a diagnostic accuracy of 84.3% (Koo et al., 2011). In the present study, the accuracy of dual-coupling model for FFRct calculation was 86.7%, the sensitivity was 66.7%, the specificity was 84.6%, the positive predictive value was 66.7%, and the negative predictive value was 91.7%. This indicates that the capacity for diagnostic prediction of FFRct calculated by the dual-coupling model is not inferior to that of conventional FFRct. Therefore, there is higher accuracy using the dual coupling model. Because clinically measured FFR is measured under real physiological conditions, we introduced the elasticity of the blood vessel into the conventional geometric multi-scale model to take into account the interaction between blood and the artery walls. The individualization of the coronary artery model makes the parameters of the 0D model more reasonable. The sensitivity of FFR_DC_ and FFR_CFD_ in this study was 66.7%, which was lower than previous studies, because the sample size of this study was only 15, of which only three patients were FFR positive.

Patient-specific allocation methods for coronary artery flow enabled individualization of the coronary microcirculation resistance model, which matched the parameters of the 0D model to each patient. The dual coupling model appeared to have physiological significance since morphological distinctions between patients was considered. Methodologically, a physics-driven dual-coupling method as described herein can be used to simulate a more realistic coronary hemodynamic environment. In clinical application, this method can improve the accuracy of FFRct calculations and can potentially assist in guiding successful clinical percutaneous coronary intervention (PCI) surgery.

## Limitations and Future Work

There are several limitations to the techniques and methodology used in the study. For instance, we assumed that the material properties of the three-layered structure of the blood vessel wall and the plaque were uniform, using a single elastic modulus. However, the blood vessel wall is divided into three layers, with plaques, and differs between patients. We propose to further develop the modeling system to personalize each patient’s plaque type and parameters related to elasticity of the blood vessel wall.

In this study, we limited enrollment and collected clinical data for 15 patients, but this sample size is small. Additional enrollment will allow us to improve the accuracy of the dual-coupling model, and also improve the algorithm that we developed. We will continue to collect more patient cases and carry out prospective clinical trials on FFRct. Meanwhile we will conduct FFR-guided and PCI-guided double-blind trials. Prognostic analysis confirmed that dual-coupled FFRct could be applied clinically to guide the operation of PCI. In addition, all 15 patients in this study had a single stenosis, whereas this method is theoretically applicable to patients with multiple stenoses, which will be further verified at a later stage.

## Conclusion

In conclusion, a physics-driven dual-coupling model for numerical FFRct-DC calculation was established, and the results of such calculations were compared to those from a more conventional CFD-based geometric multi-scale method. This new model incorporates the influence of the elastic vessel wall on blood flow and provides reliable microvascular resistance boundary conditions for the 3D FSI model. Therefore, it more closely parallels physiological conditions, resulting in improved FFRct accuracy, and enhanced accuracy of myocardial ischemia prediction. The model may be used to non-invasively provide a more reliable recommendation for clinical PCI surgery.

## Data Availability

The original contributions presented in the study are included in the article/Supplementary Material, further inquiries can be directed to the corresponding author.
